# The Efficacy of Curcumin in Reducing Immunosuppressive States of Peripheral Blood Mononuclear Cells Extracted From Oral Squamous Cell Carcinoma Patients: An In Vitro Study

**DOI:** 10.7759/cureus.77899

**Published:** 2025-01-23

**Authors:** Prakruti Dash, Saurav Nayak, Pradipta K Parida

**Affiliations:** 1 Biochemistry, All India Institute of Medical Sciences, Bhubaneswar, Bhubaneswar, IND; 2 Otorhinolaryngology, All India Institute of Medical Sciences, Bhubaneswar, Bhubaneswar, IND; 3 ENT-Head and Neck Surgery, All India Institute of Medical Sciences, Bhubaneswar, Bhubaneswar, IND

**Keywords:** curcumin, immunotherapy, immunotolerance, oral squamous cell cancer, pd-1/pd-l1

## Abstract

Background and objectives

Prior studies have shown that patients with oral cancer overexpress programmed cell death 1 (PD-1) and programmed cell death ligand 1 (PD-L1) in cancer cells and immunocompetent lymphocytes. Current immunotherapeutic interventions include antibodies targeting PD-1/PD-L1. This observational, in vitro, cell culture-based study aimed to assess the concentrations of PD-1 and PD-L1 in the peripheral blood mononuclear cells (PBMCs) of patients with oral squamous cell carcinoma (OSCC) and compare their levels with those in healthy controls, both pre- and post-curcumin intervention. This study also compared the soluble fraction of PD-L1 in the serum of patients with that in controls. We aimed to determine a cutoff level for cell surface PD-1/PD-L1 to differentiate between patients and healthy controls, in order to identify potential targets for immunotherapy.

Methodology

Blood samples (5 mL) were collected from both controls (n=20) and patients (n=20). Of this, 2 mL was used to collect serum samples, and 3 mL was used for isolation and culture of PBMCs. Cells were analyzed pre- and post-intervention with curcumin for PD-1 and PD-L1 expression.

Results

This study provides relevant data regarding cellular and serum PD-1/PD-L1 levels in patients with OSCC, which were significantly higher than in controls. Intervention with curcumin decreased PD-L1/PD-1 levels, indicating the therapeutic efficacy of curcumin in suppressing immunotolerance in the tumor microenvironment. We also found that cell lysate PD-L1 and PD-1 had a sensitivity of 75% and specificity of 89%, with cutoff values of 0.602 and 5.53 ng/mL for PD-L1 and PD-1, respectively. The receiver operating characteristic (ROC) curve analysis determined that these markers were suitable for OSCC diagnosis and identifying the appropriate cohort for immunotherapy.

Conclusions

Our study showed that serum and PBMC lysate PD-1 and PD-L1 levels were higher in advanced cancer cases compared to patients with localized disease without metastasis. Curcumin reduced the levels of PD-1 and PD-L1 in PBMC lysates. Further studies and clinical trials are required to gain deeper insights into its utility as an effective chemo adjuvant.

## Introduction

Oral cavity cancers are among the most common malignancies in India [1]. Eastern India has a higher incidence of oral cavity cancer, predominantly due to the region’s betel-chewing habits. Recently, there has been an alarming rise in the incidence of oral cancer [2,3]. Treatment for oral cavity cancer includes several methods, depending on the cancer’s stage and grade, such as surgery, radiotherapy, and chemotherapy. However, current treatments are associated with a poor five-year survival rate, recurrence, significant side effects, and drug resistance. Experimental evidence has shown an immunosuppressive state in cancers, characterized by the excessive generation of immunosuppressive cells, such as a specialized subpopulation of T cells known as T-regulatory cells (Tregs). Tregs inhibit the activation of immune T cells and reduce the production of immune-stimulatory interleukins, thereby promoting the aggressiveness of the disease [4].

Among the latest developments in cancer therapies, immunotherapy is a promising approach focusing on various molecules acting as “control switches.” These molecules include cell surface protein receptors and their ligands, which function as immune checkpoints and downregulate immune responses [5]. Programmed cell death 1 (PD-1) is a protein found on the cell surface that acts as a receptor for its ligand, programmed cell death ligand 1 (PD-L1), which is primarily expressed on immunologically activated T lymphocytes and macrophages. PD-L1 receptor activation results in the premature destruction of cells or can increase the production of Tregs. In addition to T cells and macrophages, PD-L1 may also be overexpressed in tumor cells, thereby creating an immunosuppressive microenvironment [4]. Previous studies have shown that patients with oral cancer exhibit PD-L1/PD-1 overexpression in cancer cells and immunocompetent lymphocytes [6].

Inhibiting PD-L1 expression on T lymphocytes or cancer cells enhances immune defense against tumor cells, leading to apoptosis and thereby preventing cancer cell growth [7-9]. Curcumin (Curcuma longa) is an Indian spice widely used by the population for its anti-inflammatory and anti-infective properties [10,11]. Containing the active ingredient diferuloylmethane, curcumin is currently being evaluated in numerous in vitro and in vivo studies to determine its anti-cancer properties and its role as a chemo-adjuvant for various cancer types [12-15]. The immunomodulatory effects of curcumin have emerged as a novel area of research, focusing on its potential as an immunotherapy adjuvant [16]. Further investigation into dietary polyphenols as chemo-preventive and chemo-adjuvant agents using in vitro models is essential to enhance our understanding of the utility of these natural compounds and explore their potential as new medications with broader acceptability and limited side effects.

However, data on curcumin’s effect on PD-L1/PD-1 expression suppression are currently insufficient, and prior studies on its role as an immune modulator in oral squamous cell carcinoma (OSCC) are limited. OSCC is considered a malignancy that is relatively easy to access for treatment. Additionally, curcumin is widely used and accepted by the general public. If its role in targeting PD-L1/PD-1 molecules is established, it could be safely used as an adjuvant in combination with other immunotherapy antibodies to enhance treatment effectiveness. Any local interventional therapy that has no side effects, is easily available, and is low-cost would likely be widely accepted by patients, improving compliance. Notably, curcumin is safe, non-toxic, and highly acceptable, which will facilitate the development of immunotherapy regimens for patients with OSCC.

Therefore, this study aimed to evaluate the concentrations of PD-1 and PD-L1 in peripheral lymphocytes, correlate these levels with those in controls, and assess their changes post-curcumin intervention. Additionally, we compared the soluble fraction of PD-L1 (sPD-L1) in patient serum with that in healthy controls. We also attempted to determine the cutoff level of cell surface PD-1/PD-L1 required to differentiate patients from healthy controls. Furthermore, a potential association between PD-1/PD-L1 expression in blood lymphocytes and histopathological cancer grading (TNM stage) was investigated to identify a potential target population for immunotherapy.

## Materials and methods

Study design and setting

This in vitro cell culture-based study was conducted in the Department of Biochemistry, AIIMS, Bhubaneswar, in collaboration with the Department of Otolaryngology, AIIMS. The study cohort was classified into three groups as follows: Group A consisted of age- and sex-matched healthy individuals aged 18-60 years as the control group (n=20). Group B included clinically and histopathologically confirmed patients with OSCC in stages 1-3, with localized disease and no metastasis (n=10). Group C comprised clinically and histopathologically confirmed patients with OSCC in stage 4, characterized by advanced disease with metastasis (n=10).

Eligibility criteria and sample size

The inclusion criteria were as follows diagnosed OSCC in stages 1-3 with localized disease without metastasis and stage 4 with advanced disease and metastasis. Patients were recruited immediately after confirmation of diagnosis. None of the cases had received any therapeutic intervention. The exclusion criteria included patients with OSCC already undergoing therapy or those with oral precancerous conditions. Patients with additional autoimmune disorders were also excluded. To calculate the sample size, an alpha error of 5% and a power of 90% were used.

Data collection

For analysis, a 5 mL blood sample was collected from the controls and participants. Of this, 2 mL was used to extract the serum sample, and 3 mL was used for the isolation and culture of peripheral blood mononuclear cells (PBMCs).

PBMC Isolation and Culture

Three milliliters of blood were used to isolate PBMCs through differential centrifugation at 400 g using Hisep LSM (HiMedia Laboratories, Mumbai, India) at room temperature (15-25°C) without brake for 30 minutes. The cells were cultured in RPMI 1640 medium supplemented with 1 mM sodium pyruvate, 2 mM L-glutamine, 4.5 g glucose/L, 10 mM HEPES buffer, and 2 g/L sodium bicarbonate, along with fetal bovine serum (FBS) and antibiotics. The culture was maintained in 95% air and 5% CO_2_. Cells were passaged until they reached 80% confluence following standard animal cell culture protocols. Once confluent, the cells were extracted and divided into two portions.

The first portion was treated with 1 mL of RIPA lysis buffer (HiMedia Laboratories) to prepare cell lysates for baseline PD-1 and PD-L1 estimation, which was performed in triplicate using ELISA. The second portion was resuspended in complete RPMI medium (containing FBS and antibiotics) with 20 µmol/L curcumin for 48 hours. Curcumin, obtained from Sigma-Aldrich (St. Louis, MO), was used at this concentration based on a standardization study. Serial concentrations of curcumin (12, 20, 24, 30, and 50 µmol/L) were tested, and the effect on PD-1 and PD-L1 levels plateaued at 20 µmol/L, with the peak effect observed at the same concentration. After 48 hours of incubation with curcumin, the cells were extracted and treated with 1 mL of RIPA lysis buffer. Cell lysates were then analyzed in triplicate for PD-1 and PD-L1 levels using ELISA. The resulting values for PD-1 and PD-L1 were expressed in ng/mL.

Statistical analysis

Statistical analysis was conducted using IBM SPSS Statistics v26 (IBM Corp., Armonk, NY). Data were presented as median [interquartile range (IQR)] and counts (%). The Mann-Whitney U test was applied to compare independent groups, while pre-post comparisons were assessed using the Wilcoxon signed-rank test. A receiver operating characteristic (ROC) curve analysis was performed to evaluate the diagnostic efficacy of the tests and their ratios. The cutoff value was determined using the Youden Index, and sensitivity, specificity, and the area under the curve (AUC) were derived from the ROC curve.

## Results

**Figure 1:**
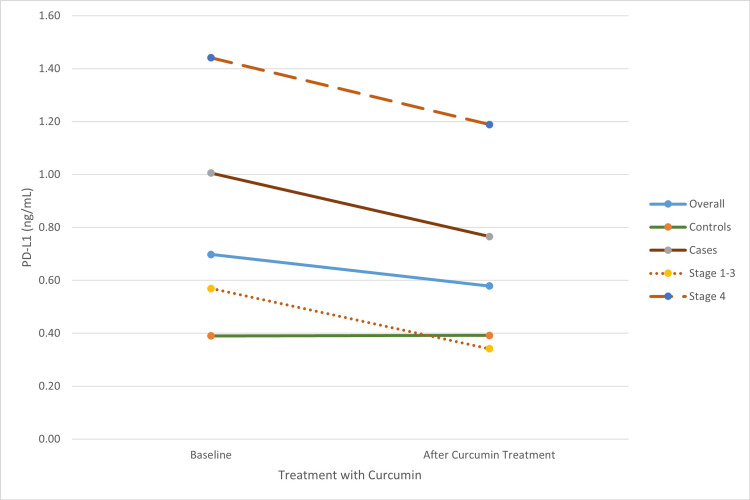
Change in cell lysate PD-L1 levels post treatment with curcumin PD-L1: programmed death-ligand 1

**Figure 2:**
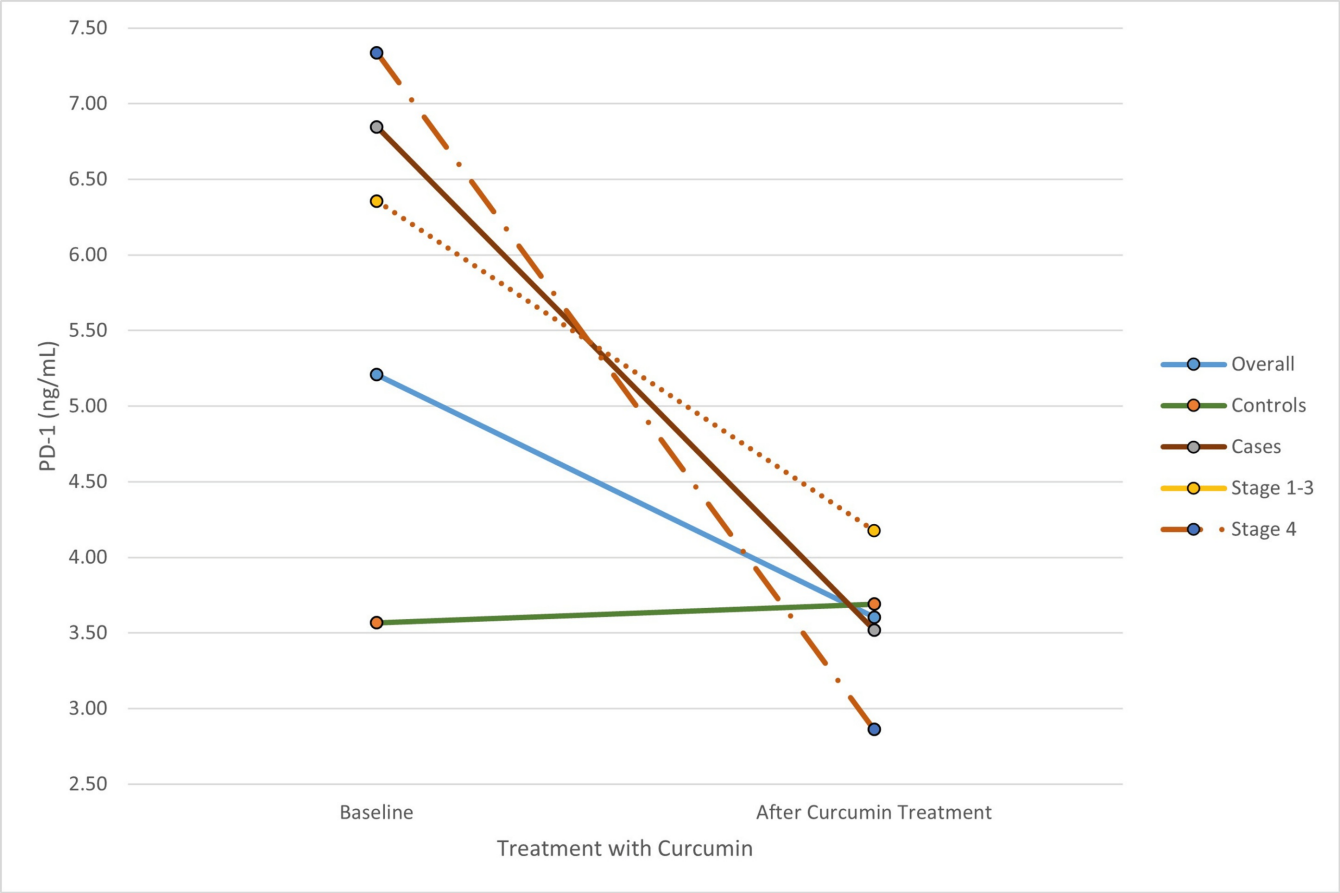
Change in cell lysate PD-1 levels post treatment with curcumin PD-1: programmed cell death protein 1

**Figure 3:**
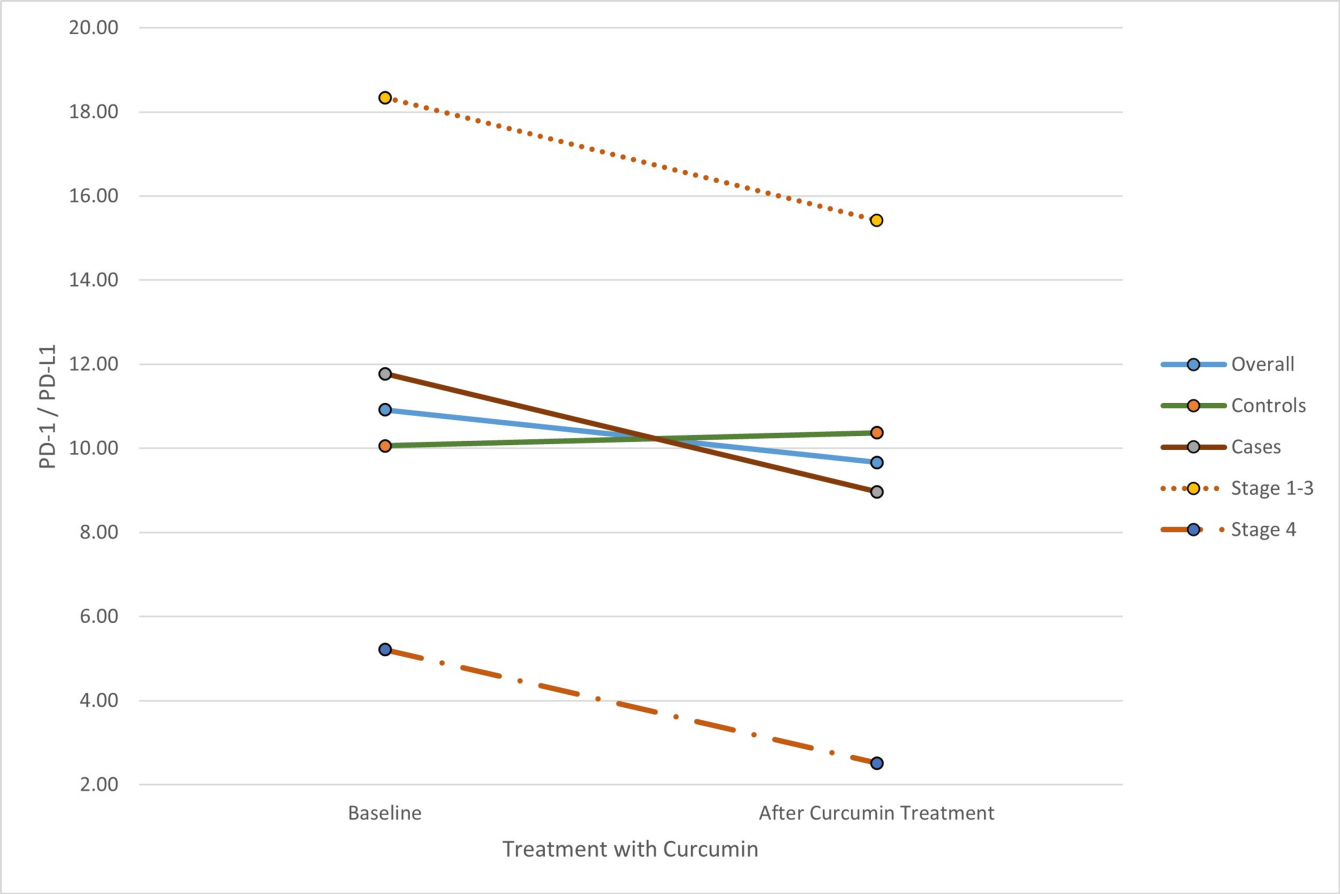
Change in cell lysate PD-1/PD-L1 levels post treatment with curcumin PD-1: programmed cell death protein 1; PD-L1: programmed death-ligand 1

Serum PD-L1 (sPD-L1) levels were significantly higher in oral cancer cases compared to controls, with a median value of 0.15 ng/mL in controls vs. 0.22 ng/mL in cases. Both PD-L1 and PD-1 levels were significantly elevated in PBMC cell lysates from cancer cases compared to controls before treatment. The median PD-L1 value in PBMC lysates was 0.37 ng/mL in controls and 1.18 ng/mL in cases. Following intervention with 20 µmol/L, the decrease in PD-1 and PD-L1 levels was more pronounced in patients than in healthy controls. Among the patient groups, the reduction in PD-L1 and PD-1 levels after the intervention was most significant in stage 4 cases (Group C) compared to stage 1-3 cases (Group B) (Table 1, Figure 1, Figure 2). Additionally, the PD-1 to PD-L1 ratio proportionally decreased post-intervention compared to the baseline ratio in cases, with the most significant decrease observed in stage 4 cases in Group C (Figure 3).

**Table 1:** Comparison of baseline and post-treatment PD-L1 and PD-1 levels in various groups by Wilcoxon signed-rank test P-value <0.05 is considered significant PD-1: programmed cell death protein 1; PD-L1: programmed death-ligand 1; Tx: treatment

Population	Difference between baseline and post-curcumin levels	P-value
Overall study population	PD-L1 (Tx – base)	0.003
	PD-1 (Tx – base)	0.001
	PD-1 to PD-L1 ratio (Tx – base)	0.307
Controls	PD-L1 (Tx – base)	0.968
	PD-1 (Tx – base)	0.794
	PD-1 to PD-L1 ratio (Tx – base)	0.575
Cases	PD-L1 (Tx – base)	<0.001
	PD-1 (Tx – base)	<0.001
	PD-1 to PD-L1 ratio (Tx – base)	0.037
Stage 1-3	PD-L1 (Tx – base)	0.017
	PD-1 (Tx – base)	0.007
	PD-1 to PD-L1 ratio (Tx – base)	0.508
Stage 4	PD-L1 (Tx – base)	0.005
	PD-1 (Tx – base)	0.005
	PD-1 to PD-L1 ratio (Tx – base)	0.007

sPD-L1 had a cutoff value of 0.165 ng/mL, with a sensitivity of 55% and a specificity of 100%. The sensitivity and specificity of PBMC cell lysate PD-1 and PD-L1, with cutoff values of >5.53 ng/mL and >0.602 ng/mL respectively, were 75% and 100%. These results suggest that PBMC cell lysates may serve as better markers for assessing PD-1 and PD-L1 in patients with oral cancer (Table 2). 

**Table 2:** Cut-off value determination for oral Ca cases based on serum and lysate values FN: false negative; FP: false positive; NPV: negative predictive value; PD-1: programmed cell death protein 1; PD-L1: programmed death-ligand 1; PPV: positive predictive value; TN: true negative; TP: true positive; Tx: treatment

Variable	Cut-off value	Youden’s J value	TP	TN	FP	FN	Sensitivity	Specificity	PPV	NPV
Serum PD-L1	>0.165	0.55	11	20	0	9	55%	84%	100%	69%
Lysate PD-L1	>0.602	0.75	15	20	0	5	75%	85%	100%	80%
Serum to lysate ratio PD-L1	>0.561	0.35	11	16	4	9	55%	80%	73%	64%
Lysate PD-1	>5.53	0.75	15	20	0	5	75%	85%	100%	80%
Lys PD-1 to PDL-1	>22.39	0.20	4	20	0	16	20%	82%	100%	56%

**Figure 4:**
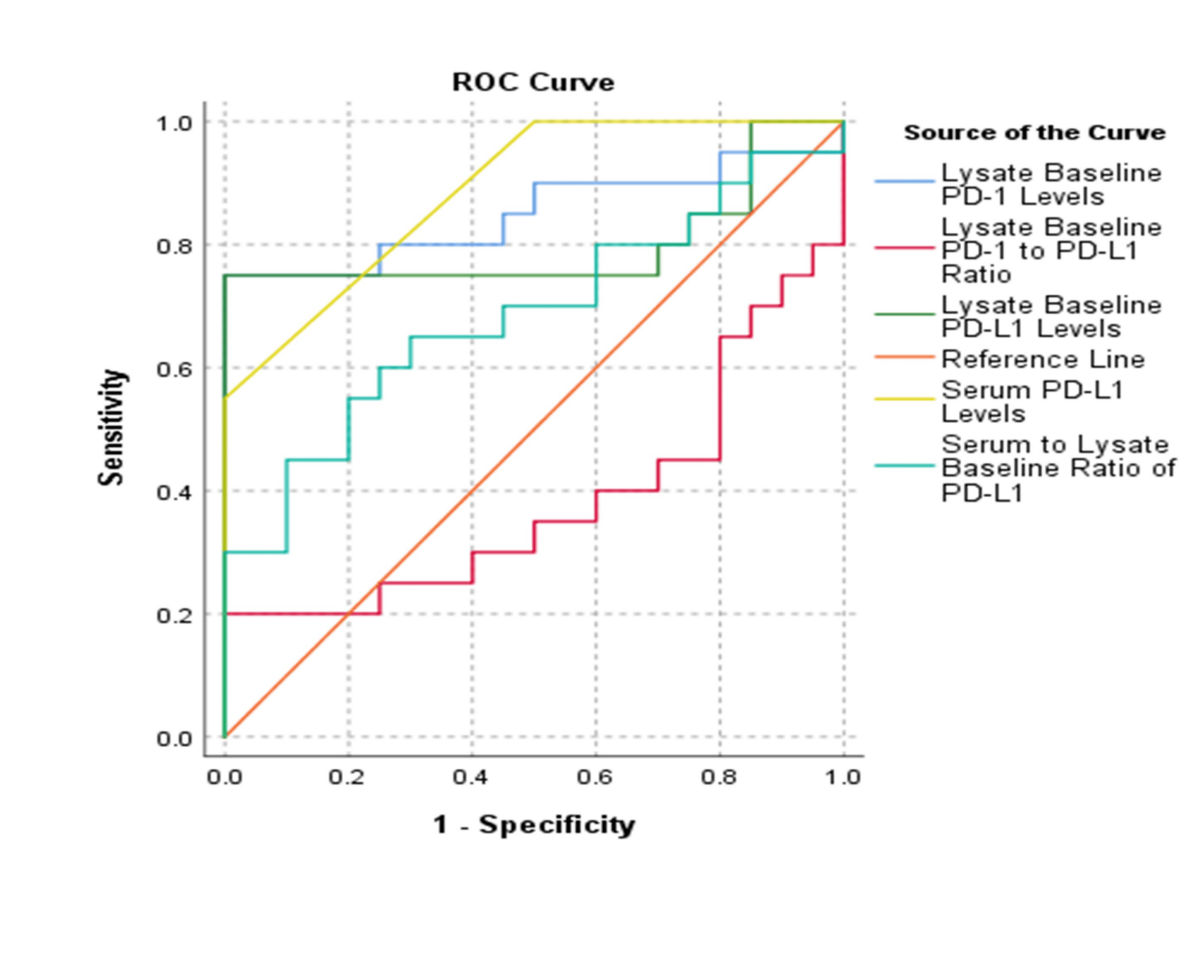
ROC curve analysis for diagnostic power of PD-1 and PD-L1 PD-1: programmed cell death protein 1; PD-L1: programmed death-ligand 1; ROC: receiver operating characteristic

The AUC for sPD-L1 and cell lysate PD-1 and PD-L1 was significant within 95% confidence intervals (CIs). sPD-L1 had an AUC of 0.888, with a p-value of <0.001. Baseline PD-L1 and PD-1 in lysates demonstrated AUC values of 0.800 and 0.850, respectively, with p-values of <0.001. The serum-to-lysate baseline ratio of PD-L1 showed an AUC of 0.68, with a p-value of 0.03. In contrast, the lysate baseline PD-1 to PD-L1 ratio had an AUC of 0.38, with a p-value of 0.20, indicating limited diagnostic utility (Table 3, Figure 4). 

**Table 3:** Area under the curve for parameters P-value <0.05 is considered significant AUC: area under the curve; PD-1: programmed cell death protein 1; PD-L1: programmed death-ligand 1; Tx: treatment

Test result variable(s)	AUC	P-value	95% confidence interval	
			Lower bound	Upper bound
Serum PD-L1 levels	0.888	<0.001	0.791	0.984
Lysate baseline PD-L1 levels	0.800	<0.001	0.642	0.958
Serum to lysate baseline ratio of PD-L1	0.685	0.031	0.516	0.854
Lysate baseline PD-1 levels	0.850	<0.001	0.716	0.984
Lysate baseline PD-1 to PD-L1 ratio	0.383	0.208	0.200	0.565

## Discussion

Immunotherapy is an emerging treatment modality that uses pharmacologic "checkpoint inhibitors" to block inhibitory signal molecules, including those in the PD-1/PD-L1 pathway. This blockade triggers an immune response against tumor cells through immunologically activated cells. In this study, the effect of curcumin on the suppression of PD-L1/PD-1 expression in PBMCs was assessed. The findings provided valuable insights into cellular and sPD-L1 levels in patients with OSCC, demonstrating significantly higher PD-L1 levels in patients compared to controls. Alrehaili et al. [17] and Zhang et al. [18] similarly reported elevated sPD-L1 levels in patients with OSCC compared to healthy controls. Feng et al. [19] documented increased expression of PD-1 and PD-L1 in CD4+ and CD8+ cells in Hodgkin’s lymphoma, contributing to immune escape by cancer cells. They also highlighted the role of sPD-L1 in monitoring therapy response in Hodgkin’s lymphoma cases. Zhu et al. [20] concluded that sPD-1 and sPD-L1 are promising biomarkers for evaluating malignant tumors in the context of immunotherapy.

This study determined that PD-1 and PD-L1 levels were significantly elevated in patient-derived PBMC-induced cell lysates compared to controls before curcumin treatment, reflecting baseline levels of these markers (Table 1). Kim et al. [21] noted in their review that peripheral blood lymphocytes could serve as biomarkers to predict the outcome of PD-1/PD-L1 blockade immunotherapy in cancer patients. They also highlighted studies reporting that tumor-specific CD8+ T cells are characterized by PD-1 expression in cancers [22,23] and that PD-1+ CD8+ T cells can be used to monitor dynamic changes in tumor-specific CD8+ T cells [21]. Weber et al. [24] observed a significant increase in PD-L1 expression in the peripheral blood of patients with OSCC and lymph node metastases. They concluded that elevated PD-L1 expression in blood samples from lymph node-positive OSCC patients indicated systemic immunosuppression.

Several studies have explored pre-therapeutic levels of PD-L1 as a potential biomarker of cancer progression. Shen et al. [25], Cheng et al. [26], and Larrinaga et al. [27] reported higher baseline sPD-L1 levels in cancers such as peripheral T-cell lymphoma, hepatocellular carcinoma, and renal cell carcinoma, correlating these levels with worse prognosis. Molga-Magusiak et al. [28] similarly detected elevated baseline PD-L1 levels in the blood of patients with head and neck cancers. They reported that sPD-L1 is a promising biomarker for differentiating malignant lesions in the head and neck region and for predicting early recurrence.

The difference in PD-1 and PD-L1 levels before and after curcumin intervention was significant, with lower levels observed post-treatment. This finding underscores the inhibitory effect of curcumin on PD-1 and PD-L1, demonstrating its potential therapeutic efficacy in suppressing the immunotolerance exhibited in the tumor microenvironment of patients with OSCC. Hawaii et al. [29] reported that combining curcumin with PD-1/PD-L1 antibodies had a synergistic antitumor effect in the MC38 murine tumor model. They advocated for combining curcumin with conventional targeted therapies as a multifaceted approach for treating head and neck squamous cell carcinoma.

Liu et al. [30] found that curcumin decreases PD-1 and PD-L1/PD-L2 expression in tumors, thereby reinvigorating exhausted T cells. They also noted that curcumin reduces regulatory T cells (Tregs, CD4+CD25+FoxP3+) and TIM-3, facilitating an immune attack and tumor cell lysis. Allegra et al. [31] concluded that curcumin exerts immunomodulatory effects through various metabolic pathways. For example, it decreases Tregs, increases CD8+ immunocompetent cells, enhances IFN-γ secretion by NK cells, and downregulates NF-κB, JAK/STAT, MAPKs, and Notch-1 activities. Additionally, curcumin nano-formulations stimulate the body’s natural defense systems and reduce inflammation, further highlighting its therapeutic potential.

The decreased levels of PD-1 and PD-L1 post-curcumin intervention were significant in all patients, with a more pronounced reduction in Group C patients compared to Group B patients. The PD-1 to PD-L1 ratio also decreased proportionately post-intervention relative to the baseline ratio in patients. Similar trends have been documented in other cancers, including melanoma, breast, gastric, liver, and pancreatic cancers [32-37]. In this study, elevated PD-1 and PD-L1 expression was noted in more advanced OSCC, consistent with the findings of Fernando et al. [38], Saeed et al. [39], Cui et al. [40], and Wang et al. [41]. The observed post-curcumin reduction in PD-1 and PD-L1 levels in cell lysates supports the role of these immune checkpoint molecules in promoting immunotolerance and T-cell exhaustion. When inhibited by antibodies or phytochemicals like curcumin, these molecules become promising targets for immunotherapy [7,29,42-45].

We found that cell lysate PD-L1 and PD-1 measurements had a sensitivity of 75% and specificity of 100%, with cutoff values of 0.602 ng/mL for PD-L1 and 5.53 ng/mL for PD-1, respectively. These findings suggest that PD-L1 and PD-1 are suitable markers for OSCC diagnosis and for identifying potential immunotherapy candidates, as demonstrated by the ROC analysis. Weber et al. [24] reported a sensitivity of 81.4% and specificity of 82.9% for PD-L1 expression, with an AUC of 0.83. In this study, we determined AUCs of 0.88 and 0.80 for sPD-L1 and lysate PD-L1, respectively, aligning with their findings. sPD-L1 demonstrated 100% specificity, though its sensitivity was lower at 55% with a cutoff value of 0.165 ng/mL. The serum-to-cell lysate ratio of PD-L1 showed significant differences, indicating that the cellular level of PD-L1 was proportional to the serum level. This ratio exhibited a sensitivity of 55% and specificity of 100% at a cutoff value of 0.561, suggesting its potential role as a marker for OSCC diagnosis and prognosis.

Han et al. [46] evaluated sPD-L1 and sPD-L2 in patients with lung cancer and concluded that combined serum levels of sPD-L1, sPD-L2, and carcinoembryonic antigen (CEA) serve as accurate biomarkers for lung cancer. They also found that higher levels of sPD-L1 (>713.75 pg/mL) were associated with poor prognosis. Ancin et al. [47] similarly reported that sPD-L1 could be a useful biomarker for predicting stage, recurrence, and metastasis in patients with non-small cell lung cancer. Previously, Yang et al. [48] found that sPD-L1 is quantifiable, easy to evaluate, and a reliable marker for assessing tumor progression and therapeutic efficacy in nasopharyngeal carcinoma. Iincorvaia et al. [49], in their study on metastatic clear cell renal carcinoma, concluded through ROC curve analysis that serum levels of sPD-1 >2.11 ng/mL and sPD-L1 >0.66 ng/mL predicted a better response to nivolumab treatment.

Toledo et al. [50] studied the role of sPD-1 and sPD-L1 in neurocysticercosis and found that sPD-1 had an AUC of 0.89 (95% CI 0.72-1), with 100% sensitivity and 72.2% specificity, using a cutoff value of 71.55 ng/mL. For sPD-L1, the AUC was 0.77 (95% CI 0.55-0.98), using a cutoff value of 95.8 ng/mL, with a sensitivity of 66.7% and specificity of 78.6%. For the sPD-1:sPD-L1 ratio, the AUC was 0.94 (95% CI 0.86-1), with 100% sensitivity and 90.48% specificity, and a cutoff value of 5.67 ng/mL to predict treatment response. Fanale et al. [51] conducted a study on high-grade serous ovarian cancer and established a baseline cutoff value for sPD-L1 (>0.42 ng/mL) and PD-1 (>2.48 ng/mL), which was associated with poor clinical outcomes and decreased progression-free survival. In another study, they identified baseline cutoff values of sPD-1 (>8.1 ng/mL) and sPD-L1 (>0.7 ng/mL) as indicators of poor prognosis and shorter disease-free survival in metastatic gastrointestinal stromal tumors [52].

In this study, we quantified the PD-1 and PD-L1 levels in PBMC lysates and found a moderate proportional correlation with OSCC stages. The levels of PD-1 and PD-L1 significantly decreased post-curcumin intervention, highlighting the therapeutic efficacy of curcumin in combination with target antibodies against PD-1 and PD-L1. PBMC lysates prove to be a convenient and effective source for evaluating PD-1 and PD-L1 pre- and post-treatment as biomarkers for OSCC.

Study strengths

PD-1 and PD-L1 levels in PBMC lysates were quantified to facilitate the convenient assessment of these molecules as potential immunotherapy candidates and to monitor therapeutic responses. Curcumin, an easily accessible phytochemical, was found to be an effective immunomodulatory substance in the treatment of OSCC and may serve as a therapeutic option following proper clinical trials.

Study limitations

This study has several limitations. The expression of PD-1 and PD-L1 in PBMCs was not evaluated post-curcumin intervention, limiting the in-depth assessment of curcumin’s role in suppressing the effects of PD-1 and PD-L1 and its potential benefits as an adjuvant therapy. This is an in vitro study with a limited sample size. Hence, further studies involving clinical trials and analyzing the effect of curcumin in vivo will help validate our findings on a broader scale, thereby confirming its potential benefits in patients with OSCC.

## Conclusions

The study demonstrated the predictive role of sPD-L1 and PBMC lysate PD-1 and PD-L1 in OSCC. The serum and cell lysate levels of PD-1 and PD-L1 were higher in cancer patients and correlated with disease severity. Serum and PBMC lysate PD-1 and PD-L1 levels were higher in advanced cancer cases compared to patients with localized disease without metastasis. Curcumin reduced the levels of PD-1 and PD-L1 in PBMC lysates, with a more substantial effect in stage 4 cases with metastasis compared to localized disease and controls. Thus, curcumin may prove beneficial as an adjuvant therapy for patients with OSCC treated with immunotherapy against PD-1 and PD-L1 and this needs further studies and clinical trials for confirmation in vivo. PD-1 and PD-L1 levels in PBMC lysates are suitable markers for OSCC diagnosis and identifying potential immunotherapy candidates. These biomarkers offer a feasible and convenient method to assess OSCC and predict therapeutic responses.
